# Effects of the Tip Location on Single Piles Subjected to Surcharge and Axial Loads

**DOI:** 10.1155/2013/149706

**Published:** 2013-12-28

**Authors:** Yaru Lv, Xuanming Ding, Dubo Wang

**Affiliations:** ^1^Key Laboratory of Ministry of Education for Geomechanics and Embankment Engineering, Hohai University, 1 Xikang Road, Nanjing 210098, China; ^2^North China University of Water Resources and Electric Power, 35 Beihuan Road, Zhengzhou 450045, China

## Abstract

When applying axial load on piles subjected to negative skin friction (NSF), the yielded NSF is gradually eliminated. The process is notably influenced by the tip location (*Y*) and still a lack of understanding. This paper reports three-dimensional numerical simulations with tip locations *Y* = 1.00 pile diameter (*D*), 0.25*D*, 0.00*D*, and −1.00*D*. It is found that, against expectations, the dragload and NSF are not proportionally related to the tip location. When maximum dragload (*P*_max_) is eventually eliminated due to an axial load, there is also a negative crest of the skin friction, indicating that NSF still exists based on the criterion of the dragload reduction. The side resistance of the piles with Y = 1.00*D* and 0.25*D* is almost fully mobilised, which is demonstrated by the increment of end resistance that greatly increases with the larger axial loads. However, the side resistance of the piles with Y = 0.00*D* and −1.00*D* has a potential capacity to carry more loads with continued displacement since the increment of end resistance increases almost linearly with axial load. Therefore, when designing the pile foundation, the inclusion of the NSF should be governed by the amount of axial load to be resisted.

## 1. Introduction

Negative skin friction (NSF) due to the surrounding soil settling more than the pile has been reported by many researchers, who examined the NSF that occurs under the application of a surcharge load [1], withdrawal of the groundwater table [2], and/or soil consolidation [3]. The occurrence of NSF always induces an additional compressive force in pile shaft, which is called dragload. The caused dragload must induce additional pile settlement, which is called downdrag [4]. The location of zero skin friction is known as the neutral plane, where the pile-soil relative displacement is zero. Therefore, when designing pile foundations, it is unconservative when NSF is not considered, which can lead to serviceability problems and even eventual failures.

A number of tests have been carried out to investigate the behaviour of bored piles subjected to NSF [2, 5–8]. Previous works have been conducted in the area of dragload on piles subjected to NSF due to a surcharge load or consolidating soil, but fewer studies have been conducted on an axially loaded single pile, let alone the dragload on an axially loaded pile [8–11]. In fact, there is lack of conditions in which piles are only subjected to surcharge loads. For example, although NSF is caused by the withdrawal of the groundwater table on piles carrying a tall building, the behaviour of NSF is heavily governed by the tall building (axial load). In recent years, several centrifuge tests have been conducted on piles subjected to surcharge and axial loads. These tests focus on the applied load conditions lack of considering the effects of the tip location [2, 3, 12]. As it is well-known, the tip location significantly affects the load transfer mechanism of piles subjected to NSF [13, 14]. It also governs the occurrence and elimination of the dragload and NSF, which is still a lock of understanding.

**Figure 1:**
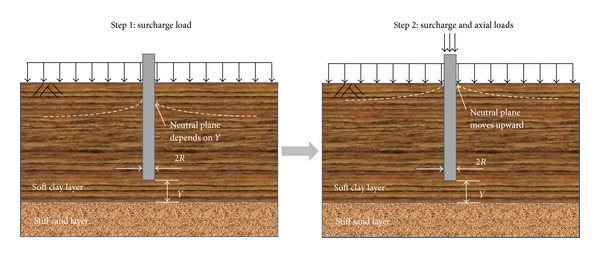
Graphical representations of a pile subjected to surcharge and axial loads.

This paper studies the effects of the tip location on single piles subjected to a surcharge load and a series of axial loads, as shown in Figure 1. When subjected to only a surcharge load (Step 1), the surrounding soil settles more than the pile, leading to a dragload and NSF. The location of the neutral plane is governed by tip location, which is abbreviated as *Y*. When pile subjected to surcharge and axial loads (Step 2), the pile starts to settle and gradually exceeds the surrounding soil, leading to NSF and dragload elimination. Therefore, as an important factor, the tip location is considered to affect the degree of dragload elimination. However, how the tip location affects the occurrence or elimination of the dragload and NSF has not been studied and fully understood. Hence, three-dimensional numerical analyses are performed on piles with tip locations *Y* = 1.00*D*, 0.25*D*, 0.00*D* and −1.00*D*, where *D* is the cross-sectional diameter of the piles and −1.00*D* means the pile tip is located into the stiff sand layer with a depth of 1.00*D*. The numerical simulation with *Y* = 1.00*D* is verified by reported measured data by Lam et al. [3].

## 2. Numerical Simulations

### 2.1. Pile Geometry, Finite Element Mesh, and Boundary and Initial Conditions

**Figure 2:**
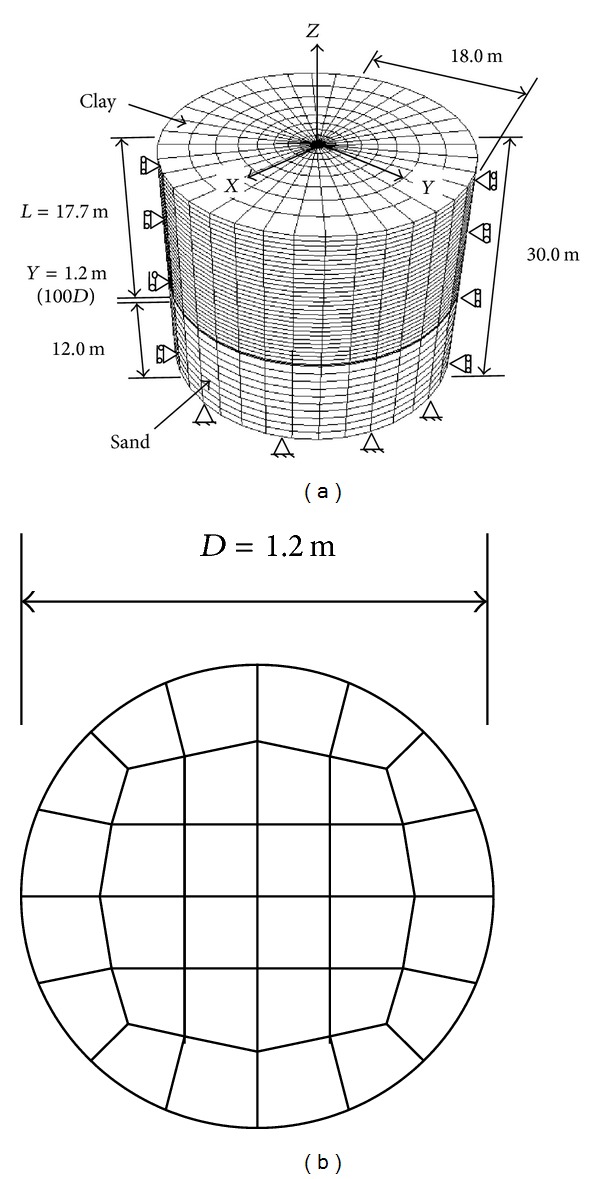
Finite element meshes adopted for: (a) the three-dimensional pile-soil system; (b) the cross-section.

Numerical analyses are carried out based on a reported centrifuge test [3] used for verification by means of a finite element programme called ABAQUS [15]. The three-dimensional element meshes and modelling scales are shown in Figure 2. Three-dimensional eight-nodded stress reduction elements are used to analyse the piles, and three-dimensional eight-nodded pore pressure elements are used to analyse the surrounding soil. Because of different tip locations, the number of elements and nodes are different. For the pile with *Y* = 1.00*D*, there are 8,960 elements and 9,785 nodes for the soil and 896 elements and 1,189 nodes for the pile. Detailed information on the elements and nodes of the remaining three pile-soil systems is summarized in Table 1. To analyse the shear stress on the interface between the pile and its surrounding soil, fine meshes are generated for the soil near the pile.

The boundary conditions are as follows: the roller supports are specified on the lateral boundary elements and the pinned supports are specified on the bottom boundary elements. The water table is kept at the ground surface, which is a drainage boundary. 45 kPa pressures are applied on top of the clay layer to simulate the surcharge load. Coupled analysis is used in this paper. Hence, at the initial conditions, the initial effective stresses are set up using the buoyant unit weight.

### 2.2. Constitutive Models and Parameters

The constitutive models and parameters for each solid are shown in Table 2. The soil properties refer to the work of Lam et al. [14]. In numerical analyses, piles are simulated to be a linear elastic material, with a Young's modulus of (*E*_*p*_ = 30 GPa). The density and void ratio are 2.7 kg/m^3^ and 0.18, respectively. The pile-soil slips are simulated using duplicated nodes to form an interface of zero thickness. The interface friction angle (*δ*) is estimated using the equation proposed by Randolph and Wroth [16] as follows:

(1) $$\begin{array}{c} \delta =\text{ta}{\text{n}}^{-1}\left[\frac{\sin\varphi^{\prime }\cos\varphi^{\prime }}{1+\text{si}{\text{n}}^{2}\varphi^{\prime }}\right]. \end{array}$$

Therefore, the interface friction coefficient of 0.32 is used in these analyses. The limiting relative shear displacement is taken as 0.005.

The soil is consisted by two layers, including 12 m sand underneath 18 m clay. The bottom sand is modeled as an elastoplastic material, with a Young's modulus of *E*_*s*_ before yielding. After yielding, the stress-strain relationships of the sand layer are described by a composite Mohr-Coulomb criterion. The sand is simulated as having a 70% relative density, corresponding to a saturated unit weight (*γ*_sat⁡_ = 19.4 kN/m^3^). Therefore, the value of *p*′ at the middle of the sand layer is approximately 100 kPa, corresponding to a maximum shear modulus of 103 MPa [17]. The soil stiffness significantly decreases with the shear strain, which is associated with foundation-related problems (typically approximately 0.1%) and is used to estimate the Young's modulus [18]. According to the empirical equation suggested by Ishibashi and Zhang [19], the shear modulus (*G*) associated with a shear strain of 0.1% is approximately 46 MPa. Therefore, the Poisson's ratio and Young's modulus of sand soil are taken as 0.3 and 120 MPa, respectively. The friction angle at the critical state (*φ*′) is 29.7°. The dilation angle is 8.3° according to the empirical equation by Bolton [20]. The initial void ratio of 0.37 of the stiff sand is back-calculated by the water content [14].

**Figure 3:**
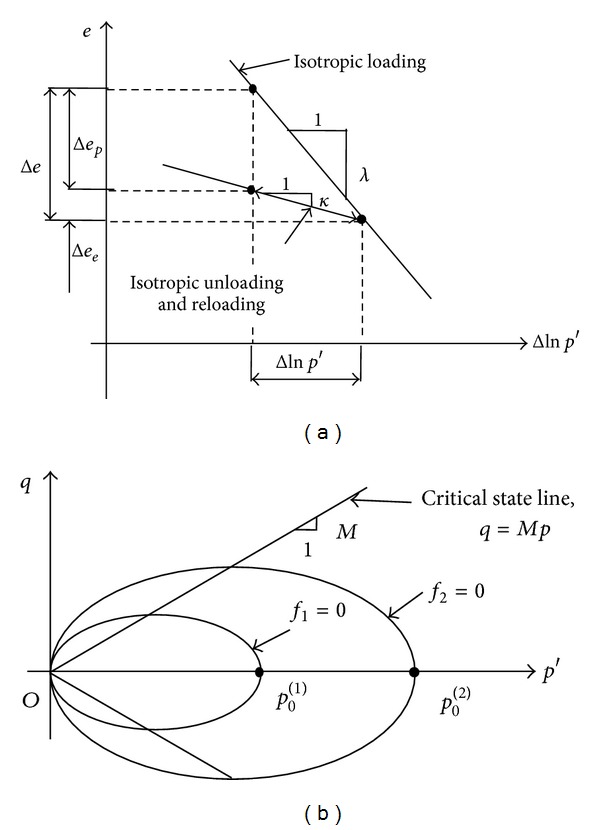
Criterion of the Cam-clay model of: (a) loading/unloading/reloading in *e*-ln*p* space; (b) yield surface on *p*′-*q* space [15].

For the top clay stratum, the failure criterion is described by the modified Cam-clay model, which was developed at Cambridge between 1960–1970 by Roscoe and Burland [21]. One of the basic characteristics of the model is non-recoverable material deformation (called plastic) after the nonlinear elastic response.  Figure 3(a) illustrates the isotropic loading/unloading/reloading on the *e*-ln⁡*p*′ plot. Experiments show that isotropic consolidation follows the isotropic loading line characterized by a material constant *λ*. Because of the elasto-plastic nature, the unloading path does not follow the consolidation loading line. Instead, the material follows an unloading line characterized by a material constant *κ*. When the material is reloaded, it usually follows the same path as unloading until the mean normal stress *p*′ exceeds the maximum *p*′ in the loading history, and it then follows the load line again. The two material constants can be obtained by the following equations:

(2) $$\begin{array}{c} \Delta e=-\lambda \Delta \ln p^{\prime },\\ \Delta e^{e}=-\kappa \Delta \ln p^{\prime }, \end{array}$$

where *e* is the void ratio, *p*′ is the mean stress, and *q* is the deviatoric stress. Because a plastic volumetric strain increment can occur only when the current stress is on the yield locus, under isotropic loading, *p*′ equals *p*_0_′. Thus, the hardening parameter *p*_0_′ is a function of *ε*_*v*_^*p*^, which is given by

(3) $$\begin{array}{c} d\varepsilon_{v}^{p}=\frac{\lambda -\kappa dp_{0}^{\prime }}{1+e_{0}p_{0}}. \end{array}$$

Figure 3(b) shows the yield surface of the modified Cam-clay model in *p*′-*q* space. The yield function of the modified Cam-clay model can be written by

(4) $$\begin{array}{c} f=M^{2}p^{\prime 2}-M^{2}p_{0}^{\prime }p^{\prime }+q^{2}=0, \end{array}$$

where *M* is the slop of the critical state line and *p*_0_′ is the value of *p*′ at the intersection of the yield locus with the *p*′ axis, where *q* = 0. The initial void ratio (*e*_0_ = 1.6) is obtained by reported measurements [3]. The values of *λ* = 0.14 and *κ* = 0.012 are specified through one-dimensional consolidation tests. Based on this test, the slop of the critical state line in *p*′-*q* space (*M*) is given as 0.98 [3].

### 2.3. Modelling Procedures

As a setting for the simulation, the piles are installed wished-in-place (the piles are placed at the desired location without drilling a hole and applying the grouting material). The details of the simulation procedures are summarized as follows.

1. Create pile-soil numerical meshes and give each part the corresponding material properties.
2. Apply the boundary conditions.
3. Establish the initial stress conditions. The geostatic step is carried out with the lateral earth pressure ratio *K*_0_ = 1 − sin*φ*′. After the geostatic step, the settlement of the ground is smaller than 10^−5^ m.
4. Apply a pressure of 45 kPa on the ground surface to simulate the surcharge load. Reveal the tip location effects on the occurrence of the dragload and NSF. This step lasts for 6.75 years for clay consolidation according to the centrifuge tests conducted by Lam et al. [14].
5. Apply the axial load (*P*) step by step with an increment of *P*/*P*_max⁡_ = 0.25 (*P*_max⁡_ is the maximum dragload which is obtained in step 4) to reveal the tip location effects on the elimination of the dragload and NSF, the variation of pile capacity, and the load transfer mechanism.

## 3. Verification of the Numerical Modelling

Tip location effects have been reported by Ng et al. [14], who mainly focus on revealing the differences between an end-bearing pile (*Y* = −1.00*D*) and a floating pile (*Y* = 0.25*D*). The results show that the neutral plane of the floating pile is located at approximately *z*/*H* = 0.75. For the end-bearing pile, the neutral plane is located near the bottom of the embedded pile length, causing NSF that almost acts on the whole pile. The result agrees with other studies [2, 7]. In the present analyses, to reveal the effects of the tip location, parametric studies are conducted on two floating piles with *Y* = 1.00*D* and 0.25*D* and two end-bearing piles with *Y* = 0.00*D* and −1.00*D*. The load condition changes from only a surcharge load to both surcharge and axial loads. The constitutive models and element meshes of the pile with *Y* = 1.00*D* are verified by means of the reported centrifuge test data [14].

### 3.1. Measured and Computed Dragload

**Figure 4:**
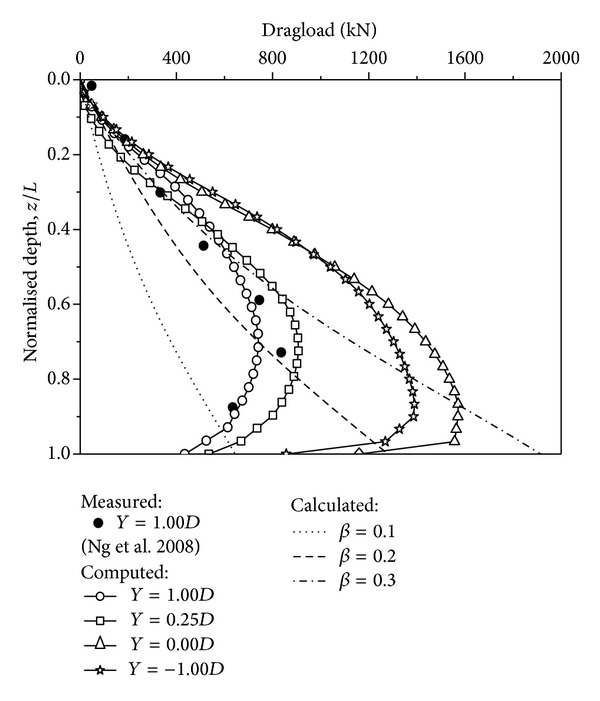
Measured and computed dragload distribution due to a surcharge load.

Figure 4 shows the measured [14] and computed dragload of the piles with tip locations *Y* = 1.00*D*, 0.25*D*, 0.00*D*, and −1.00*D*. To assist in the interpretation of the measured and computed dragload, the *β*-method is used to estimate the NSF [22–24], expressed by

(5) $$\begin{array}{c} f_{s}=\beta \sigma^{\prime }, \end{array}$$

where *f*_*s*_ and *σ*′ are the NSF mobilised along the pile shaft and the effective overburden pressure, respectively. In soft clay, the value of *β* is suggested as 0.25. Meyerhof [25] constructed a comprehensive summary on 20 sets of field data on *β* values ranging from 0.18 to 0.35. Using (5), reference lines using three *β* values (0.1, 0.2, and 0.3) are calculated and plotted in the figure for comparison.

The measured and computed dragload increases at the upper depth and then decreases under the neutral plane. On the top of the neutral plane, the measured dragload [14] for the pile with *Y* = 1.00*D* can be described by *β* = 0.26, and the computed dragload can be estimated by *β* = 0.30. It should be noted that the *β*-method only gives a reasonable estimation of the dragload at the upper bound. It cannot take into account the reduction of the dragload at the lower bound.

The measured and computed maximum dragloads of piles with different tip locations are summarized in Table 3. Because the stiffness of the bottom sand is simulated substantially higher than that of the clay layer, the dragload on the floating pile increases with decreasing tip location. However, because the downdrag decreases with decreasing tip location, the dragload of the pile with *Y* = −1.00*D* is smaller than that of the pile with *Y* = 0.00*D*. The measured and computed maximum dragload of the pile with *Y* = 1.00*D* are 849 kN and 741 kN, respectively. The measured maximum dragload is 15% larger than the computed maximum dragload, indicating agreement between the numerical simulation and the centrifuge test. The computed maximum dragload of the piles with tip locations *Y* = 0.25*D*, *Y* = 0.00*D*, and *Y* = −1.00*D* are 907 kN, 1571 kN, and 1390 kN, respectively, which are 122%, 212%, and 188% that of the pile with *Y* = 1.00*D*. In other words, the maximum dragload increases by 112% as the tip location ranges from *Y* = 1.00*D* to *Y* = 0.00*D*. Hence, the tip location should be as large as possible when floating piles are subjected to only a surcharge load. A large tip location can reduce the dragload and compression efficiently.

### 3.2. Measured and Computed Skin Friction

**Figure 5:**
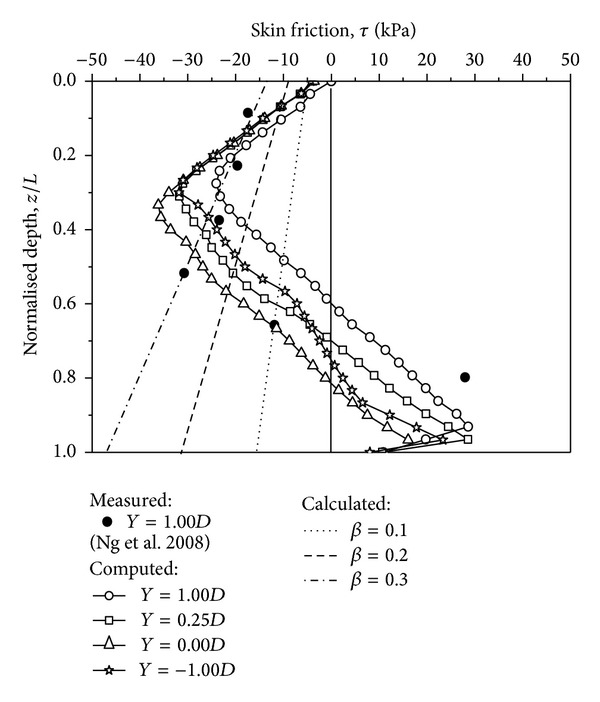
Measured and computed skin friction distribution due to a surcharge load.

The measured [14] and computed skin friction (*τ*) are illustrated in Figure 5. At the upper depth, the surrounding soil settles more than the pile, dragging the pile to settle with the surrounding soil. At the deeper depth, the pile tip inserts into the stiff sand layer, resulting in a larger displacement of the pile tip than its surrounding soil. Neutral planes exist at the interface of the downward and upward relative displacements. Hence, an important characteristic of the skin friction, the neutral plane, is yielded. The computed neutral plane is located at a depth of *z*/*H* = 0.60 and 0.71 for the floating piles with *Y* = 1.00*D* and 0.25*D*, respectively. Furthermore, the computed neutral planes are located at depths of *z*/*H* = 0.82 and 0.75 for the end-bearing piles with *Y* = 0.00*D* and −1.00*D*, respectively. This result indicates that the computed neutral plane shifts downward as the tip location decreases. However, when the pile tip is located in the stiff sand layer, the relative displacement between the pile and its surrounding soil is controlled, resulting in a smaller skin friction and a shallower neutral plane for the pile with *Y* = −1.00*D* compared to the pile with *Y* = 0.00*D*. As a reference, the measured neutral plan is located at *z*/*H* = 0.73 [14], indicating that the measured and computed results are found to be satisfactory.

Four piles have a similar trend that the computed NSF peaks at depth approximately *z*/*H* = 0.3. Meanwhile, the computed NSF peaks at depth approximately *z*/*H* = 0.5. Additionally, the measured positive skin friction (PSF) does not have a peak value, but the computed PSF peaks at about *z*/*H* = 0.9. This is because it is difficult to install the strain gauge continuously to monitor the dragload and skin friction in the centrifuge test. Hence, there is lack of measured data near the pile tip, thus no peak value for the measured PSF. In this analysis, both the floating pile (*Y* = 1.00*D* and 0.25*D*) and end-bearing pile (*Y* = 0.00*D* and −1.00*D*) have PSF when subjected to the surcharge load. Each pile tip of four cases still settles more than its corresponding surrounding soil for the end-bearing piles. This phenomenon may be caused by two reasons. First, the relative stiffness between the clay layer and the sand layer is limited in this analysis. Second, the sliding effect of the pile-soil interface is used and affects the behaviour of skin friction, as discussed by Jeong et al. [13].

For reference, the calculated skin frictions by means of the *β*-method (*β* = 0.1, 0.2, and 0.3) are also shown in the same figure. As discussed in the dragload, the measured NSF can be described by *β* = 0.26 for the shallower depth. However, it cannot reveal the nonlinear variation of skin friction, especially for floating piles because the calculated skin friction varies linearly along the depth. Therefore, the *β*-method is more reasonable for estimating the NSF of end-bearing piles because they have a limit PSF.

## 4. Effects of the Tip Location on Piles Subjected to a Surcharge Load

**Figure 6:**
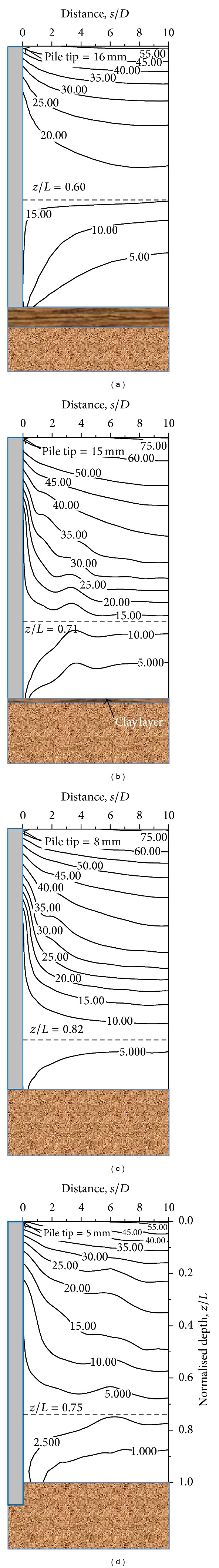
Settlement contour of the soil surrounding a pile with tip location: (a) *Y* = 1.00*D*, (b) *Y* = 0.25*D*, (c) *Y* = 0.00*D*, and (d) *Y* = −1.00*D*.

As is well known, the occurrence of PSF and NSF is governed by the relative displacement between the pile and its surrounding soil. To show the effects of the tip location directly, the settlement of the surrounding soil in relation to the surface contour is plotted in Figures 6(a), 6(b), 6(c), and 6(d) for piles with tip locations *Y* = 1.00*D*, 0.25*D*, 0.00*D*, and −1.00*D*, respectively. When a surcharge load is applied to the surrounding soil, the displacement of the surrounding soil is larger than that of the pile at the upper depth. The negative relative displacement induces a downward-acting NSF. With increasing depth, most of the applied surcharge load is redistributed from the surrounding soil to the pile through the pile-soil interface. The tip of the floating pile settles more than its surrounding soil because of the lower stiffness of the bottom soil (soft clay layer), inducing obvious upward-acting PSF. However, the tip of the end-bearing pile has limited settlement because of the higher stiffness of the bottom soil (stiff sand layer), leading to slight upward-acting PSF. Therefore, there is an obvious intersection between upward-acting PSF and downward-acting NSF, which is governed by tip location.

Against expectations, the settlement of the surrounding soil does not increase proportionally with decreasing tip location, especially for end-bearing piles. When encountering only a surcharge load, both the surrounding soil and the pile with *Y* = −1.00*D* settles less than the pile-soil system with *Y* = 0.00*D*, leading to a phenomenon in which the neutral plane of the pile with *Y* = −1.00*D* is shallower than that of the pile with *Y* = 0.00*D*.

As summarized in Table 3, the computed maximum soil settlements are 55 mm, 75 mm, 75 mm, and 55 mm for piles with tip locations of *Y* = 1.00*D*, 0.25*D*, 0.00*D*, and −1.00*D*, respectively. On the other hand, the corresponding pile settle 16 mm, 15 mm, 8 mm, and 5 mm, respectively. It is concluded that the dragload and skin friction are heavily influenced by the relative pile-soil displacement. The larger the relative pile-soil displacement is, the larger the induced dragload and skin friction are for floating piles before yielding. From these contours, these contours, the locations of the neutral plane can be clearly illustrated. It is the zone surrounded by the downward settlement curve and the upward settlement curve.

A single pile influences the zone approximately 6D away from the pile [7]. Because of the drag effects induced by the pile, the settlement of the surrounding soil increases from the center to the far field. In this analysis, three piles with *Y* = 1.00*D*, 0.25*D*, and 0.00*D* have a sphere of influence smaller than 6*D*, as shown in the contours. However, the pile with *Y* = −1.00*D* has a larger sphere of influence, approximately 8*D* far away from the pile, which may be caused by the embedded pile tip. For this case, the upper part of this pile is dragged by its surrounding soil, but the pile tip is difficult to insert into the stiff sand layer. Therefore, a large shear stress develops adjacent to the pile, resulting in vertical soil arching along the pile shaft. At the zone outside 8*D*, the settlement curve is almost a horizontal line at a given depth.

## 5. Effects of the Tip Location on Piles Subjected to Surcharge and Axial Loads 

### 5.1. Measured and Computed Pile-Load Settlement

**Figure 7:**
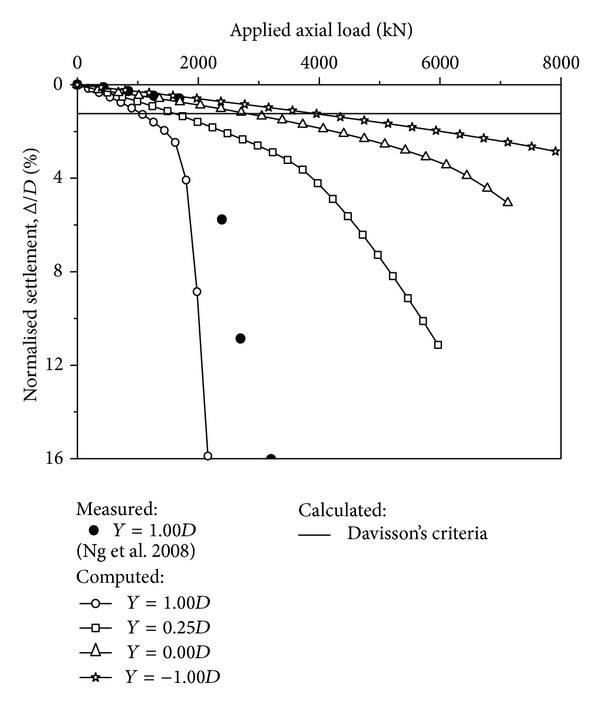
Measured and computed pile head settlement due to axial loads.

The settlement of a floating pile is more significant than that of an end-bearing pile [2, 6–8, 26]. The computed pile head settlements against an axial load are shown in Figure 7. The settlement is normalised by the pile diameter. The reported measured data (pile with *Y* = 1.00*D*) are taken into account and plotted for verification [12]. To reveal the process of dragload elimination, 20 steps of the axial load are applied to each pile with increments of 0.25 *P*_max⁡_; that is, *P*/*P*_max⁡_ = 0.25, 0.50, 0.75, 1.00, 1.25, 1.50, 1.75, 2.00, 2.25, 2.50, 2.75, 3.00, 3.25, 3.50, 3.75, 4.00, 4.25, 4.50, 4.75, and 5.00.

When designing deep foundations subjected to NSF, serviceability instead of the ultimate state usually becomes the governing factor. Because NSF is usually associated with a large settlement even with a small dragload (such as the pile with *Y* = −1.00*D* being studied), excessive settlement could develop on shaft bearing piles with pile tips in compressive soils, which is true for the *Y* = 1.00*D* case being studied. Davisson's criterion [27] gives the method for estimating the allowable loads for a pile. It can be written by

(6) $$\begin{array}{c} \Delta =\frac{PL}{AE_{p}}+\frac{D^{\prime }}{120}+4, \end{array}$$

where *L* is the pile length (mm), *A* denotes the cross-sectional area of the pile (mm^2^), *E*_*p*_ denotes Young's modulus for the material of the pile (kN/mm^2^), and *D*′ denotes the least lateral dimension of the pile (mm). Using this criterion, the normalised settlement of the piles being studied is Δ = 1.24*D* without varying with tip location. Therefore, the allowable load, also the pile capacity in this study, is 1728 kN for the stated centrifuge test. For the computed load-settlement curve, the allowable load for the pile with *Y* = 1.00*D* is 1080 kN, which is approximately 63% that of the measured results. Both the measured and computed load-settlement curves have a similar trend. The pile-soil system yields at an axial load of approximately 1600 kN. When the axial load is smaller than 1600 kN, an almost linear variation of the settlements is induced, indicating the elastic compression that occurs in soils. When the axial load is larger than 1600 kN, both the measured and computed curves are far too steep to be taken as the serviceability condition. It is confirmed that the numerical simulations, including the constitutive models and element meshes, are reasonable.

As expected, the pile settlement decreases with the decreasing tip location. Two floating piles with tip locations *Y* = 1.00*D* and 0.25*D* have obvious elastic compression stages, with 1600 kN and 3400 kN being the yield axial loads, respectively. However, two end-bearing piles have no obvious yielding axial load until the last axial load step of, *P*/*P*_max⁡_ = 5.00. Taking Davisson's criteria as the basis, the allowable loads of the piles with tip locations *Y* = 0.25*D*, 0.00*D*, and −1.00*D* are 1625 kN, 2822 kN, and 3955 kN, respectively, which are 150%, 261%, and 366% of that of the pile with *Y* = 1.00*D*, respectively. It is concluded that the tip location controls pile settlement and improve pile capacity efficiently. From *Y* = 1.00*D* to *Y* = −1.00*D*, the pile length (material waste) is increased by 14%, but the pile capacity can be improved by 266%. For this reason, the end-bearing pile is recommended when subjected to the axial loads.

### 5.2. Effects of the Tip Location on Dragload Reduction and the End Resistance Increment

**Figure 8:**
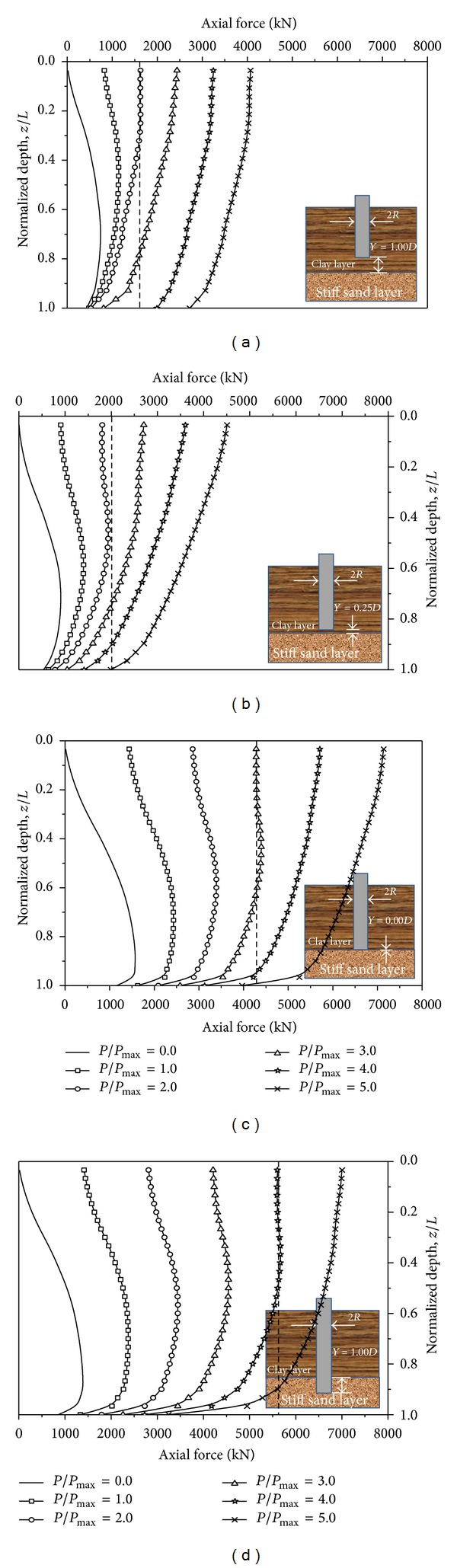
Computed variation of the axial force of a pile with tip location: (a) *Y* = 1.00*D*, (b) *Y* = 0.25*D*, (c) *Y* = 0.00*D*, and (d) *Y* = −1.00*D*.

As the load component changes from only a surcharge load to both surcharge and axial loads, the variations of the axial force for the piles with *Y* = 1.00*D*, 0.25*D*, 0.00*D*, and −1.00*D* are illustrated in Figures 8(a), 8(b), 8(c), and 8(d), respectively. As stated, the surcharge load yields the downward settlement of the surrounding soil, causing the piles to settle. Hence, the surrounding soil settles more than the pile, leading to a peak value of the dragload at the depth of the neutral plane. With different tip locations, the magnitudes of the dragload and the locations of the neutral plane are different.

As the axial load increases, a pile gradually settles faster than its surrounding soil. The position of the maximum dragload shifts upward, eliminating the dragload and NSF. Both the floating pile and the end-bearing pile have the same trend in that the maximum dragload eventually disappears, and is replaced by the maximum axial force at the pile head. However, it should be noted that the axial force along the pile shaft is not simply the sum of the dragload that is caused by the surcharge load and the axial force that is caused by the axial load. Under this load condition, the slope of the axial force and the proportion of the end resistance differ for different tip locations, which will be discussed later.

**Figure 9:**
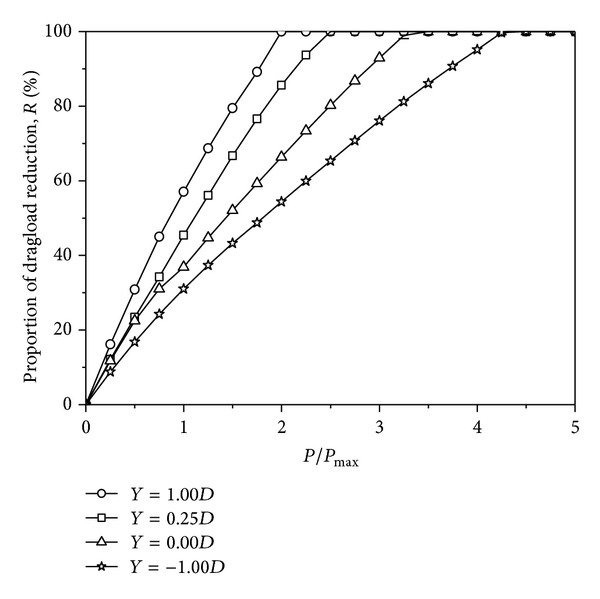
Computed maximum dragload reduction under surcharge and axial loads.

To describe the variation of the axial force from only a surcharge load to surcharge and axial loads, one coefficient, that is, the maximum dragload reduction, is defined. Jeong et al. [13] quantified the dragload reduction caused by the application of an axial load by using the following simple equation:

(7) $$\begin{array}{c} R=\frac{P_{\max}-P_{\max,\text{axial}}}{P_{\max}}\times 100\%, \end{array}$$

where *R* is the rate of maximum dragload reduction; *P*_max⁡_ is the maximum dragload before the application of an axial load; and *P*_max⁡,axial_ is the maximum dragload at a given axial load. The reduction of the maximum dragload for each pile is summarized in Table 4 and shown in Figure 9. It can be observed that the reduction of the maximum dragload varies almost linearly. The larger the tip location is, the more easily the dragload is completely eliminated. The maximum dragload reduction reaches 100% at approximately *P*/*P*_max⁡_ = 2.0, 2.5, 3.5, and 4.5 for the piles with *Y* = 1.00*D*, 0.25*D*, 0.00*D*, and −1.00*D*, respectively. This result coincides with Jeong et al. [27], who reported that an axial load of approximately 1.25 to 3.25 *P*_max⁡_ is required to eliminate dragload completely. For an end-bearing pile, especially a pile with the tip located in stiff bottom soil (as with the pile with *Y* = −1.00*D* being studied), although the magnitude of the dragload is limited due to the surcharge load, it is difficult to eliminate completely when subjected to axial loads.

**Figure 10:**
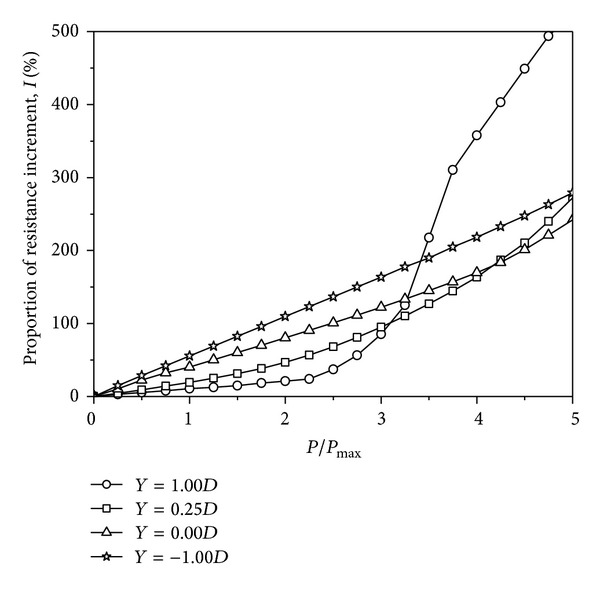
Computed end resistance increment under surcharge and axial loads.

Furthermore, to describe the variation of the end resistance from only a surcharge load to both surcharge and axial loads, the other coefficient, that is, the end resistance increment, is defined as follows:

(8) $$\begin{array}{c} I=\frac{q_{b}-q_{b,\text{axial}}}{q_{b}}\times 100\%, \end{array}$$

where *I* is the base stress increment, *q*_*b*_ is the dragload at the pile tip before the application of an axial load, and *q*_*b*,axial_ is the axial force at the pile tip under a given axial load. The end resistance increment for each pile is summarized in Table 4 and shown in Figure 10. For the piles with *Y* = 1.00*D* and 0.25*D*, the increments of the base stress increase nonlinearly with the axial load and at an increasing rate. For the pile with *Y* = 1.00*D*, the end resistance heavily contributes to the pile capacity, which may be caused by the full mobilization of the side resistance at a larger axial load. For the piles with *Y* = 0.00*D* and −1.00*D*, the increments of the base stress increase almost linearly with the axial load. As stated, the maximum dragload is eliminated completely at *P*/*P*_max⁡_ = 2.0, 2.5, 3.5, and 4.5 for the piles with *Y* = 1.00*D*, 0.25*D*, 0.00*D*, and −1.00*D*, respectively. Under the corresponding axial load, the increment of each end resistance is approximately 21%, 68%, 145%, and 248%, respectively. It is confirmed that the end resistance plays an important role for end-bearing piles before its dragload is eliminated completely.

From only a surcharge load to surcharge and axial loads, the variations of those two coefficients are governed by stress transfer mechanisms between each pile and its surrounding soil. Under a surcharge load, the surface load is carried by the soil and then transferred to the pile by means of NSF. Under both surcharge and axial loads, the load is carried by the pile and then transferred to the soil by means of the pile-soil interface. Therefore, both the maximum dragload reduction and the base stress increment result from the variation of the stress condition. Thus, when designing the pile foundation, the dragload should not be added to the loads from the structure and the capacity value should not be reduced by the dragload. The inclusion of NSF in the foundation design should be governed by the amount of axial load to be resisted. Exceeding the *P*/*P*_max⁡_ value that the dragload is eventually eliminated, the additional compressive force should not be taken into account. Although the end-bearing pile has a higher pile capacity, the procedure of dragload elimination is difficult.

### 5.3. Effects of the Tip Location on Skin Friction

**Figure 11:**
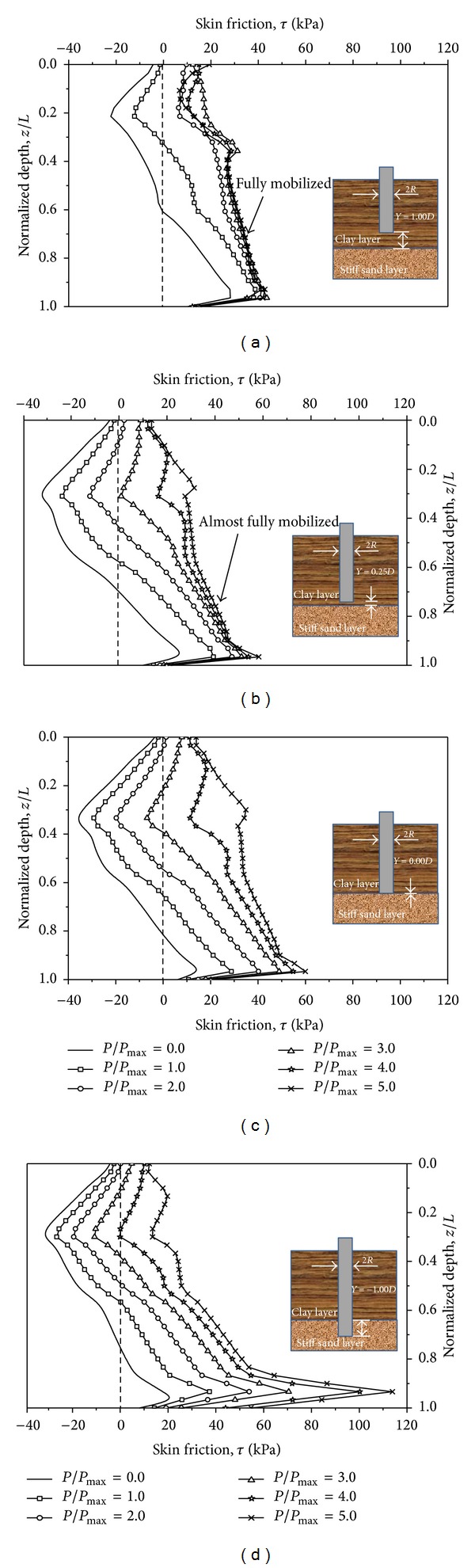
Computed variation of the skin friction of a pile with tip location: (a) *Y* = 1.00*D*, (b) *Y* = 0.25*D*, (c) *Y* = 0.00*D*, and (d) *Y* = −1.00*D*.

The computed variations of the skin friction are shown in Figures 11(a), 11(b), 11(c), and 11(d) for the piles with *Y* = 1.00*D*, 0.25*D*, 0.00*D*, and −1.00*D*, respectively. As stated in dragload, when subjected to a surcharge load only, the positions of the neutral plane yield at *z*/*L* = 0.60, 0.71, 0.82, and 0.75, respectively. When subjected to both surcharge and axial loads, the neutral plane moves upward. At a certain axial load, there are two neutral planes for the skin friction. This is caused by the relative pile-soil displacement. With increasing axial load, the pile first settles and then drags the surrounding soil to settle. This mechanism contradicts the stated mechanism of piles subjected to a surcharge load only. When the axial load is large enough, the neutral plane disappears. In this analysis, the neutral plane disappears at *P*/*P*_max⁡_ = 2.0, 3.0, 4.0, and 5.0 for the piles with *Y* = 1.00*D*, 0.25*D*, 0.00*D*, and −1.00*D*, respectively. The load steps follow these values for the maximum dragload elimination.

For piles with different tip locations, the variation of the skin friction encounters different processes. For floating piles, the skin friction of the piles with *Y* = 1.00*D* and 0.25*D* almost does not increase with increasing axial load after *P*/*P*_max⁡_ = 3.0 and 4.0, respectively. This indicates that the side resistance of each pile has almost been fully mobilised under the corresponding load step. This is the explanation of Figure 10, in which the increment of the end resistance greatly increases with the applied axial load after *P*/*P*_max⁡_ = 2.0 and 3.0 for the piles with *Y* = 1.00*D* and 0.25*D*, respectively. For end-bearing piles, the skin friction still proportionally increases with the axial load, which agrees with the increment of the end resistance in Figure 10. This phenomenon means that the side resistance has not been fully mobilised for end-bearing piles because of limited pile-soil relative displacement. In other words, the side resistance can be improved by sacrificing further settlement.

There is an unexpected occurrence that NSF due to the surcharge load is not fully eliminated when the maximum dragload disappears. Four piles have the same phenomenon that there is a negative crest of the skin friction, although the maximum dragload is fully eliminated. Therefore, when designing pile foundations, it should be noted that the NSF still exists based on the criterion of the maximum dragload elimination.

## 6. Conclusions

Three-dimensional numerical analyses are performed to investigate the effects of the tip location on piles subjected to surcharge and axial loads by considering the occurrence of the dragload and NSF, the elimination of the dragload and NSF, the variation of the pile capacity, and the load transfer mechanisms. The back-analysis of the pile with *Y* = 1.00*D* is verified by the reported centrifuge test. The measured and computed results are satisfactory, indicating the reasonability of the constitutive models and element meshes. As the basis of those numerical analyses, several conclusions are drawn as follows.

1. As expected, the smaller the tip location is, the larger the dragload and skin friction are yielded for floating piles that the pile tip does not insert into the stiff funding soil. This is because the relative pile-soil displacement increases with decreasing tip location. Against expectations, the deeper the tip location is, the smaller the dragload and skin friction are for end-bearing piles that pile tip insert into the stiff funding soil. This effect is observed because the pile settlement is controlled by stiff funding sand. Hence, as the basis of this study, in order to reduce the dragload and the compression of pile shaft efficiently, the tip location of the floating pile should be as large as possible when meeting the requirement of the bearing capacity but the pile tip of the end-bearing pile should be located as deep into the bottom soil as possible.
2. The tip location plays an important role in controlling the pile settlement and improving pile capacity. From *Y* = 1.00*D* to *Y* = −1.00*D*, the pile length (material waste) is increased by 14%, but the pile capacity is improved by 266%. For this reason, the end-bearing pile is recommended when subjected to axial loads without surcharge load or when the surcharge load has been fully eliminated.
3. When the load condition changes from a surcharge load to surcharge and axial loads, the developed dragload can be eliminated completely at *P*/*P*_max⁡_ = 2.0, 2.5, 3.5, and 4.5 for the piles with *Y* = 1.00*D*, 0.25*D*, 0.00*D*, and −1.00*D*, respectively, and the corresponding NSF is fully eliminated at *P*/*P*_max⁡_ = 2.0, 3.0, 4.0, and 5.0, respectively. There is a negative crest of the skin friction, although the maximum dragload is fully eliminated. In other words, it should be noted that when designing a pile foundation, NSF still exists based on the criterion of the maximum dragload elimination.
4. At *P*/*P*_max⁡_ = 2.0 and 3.0, the skin friction of the floating piles with *Y* = 1.00*D* and 0.25*D* almost does not increase with increasing axial load, respectively. This indicates that the side resistance of the floating piles is almost fully mobilised, as illustrated by the end resistance increment, which greatly increases with the axial load after *P*/*P*_max⁡_ = 2.0 and 3.0, respectively. However, from *P*/*P*_max⁡_ = 0.0 to *P*/*P*_max⁡_ = 5.0, the skin friction of the end-bearing pile still increases proportionally with the axial loads. This finding means that the side resistance of the end-bearing pile can carry more axial load by sacrificing further settlement since the end resistance increment almost linearly increases with the applied axial load.
5. Therefore, when designing the pile foundations, the inclusion of NSF in the foundation design should be governed by the amount of applied axial load to be resisted and heavily influenced by the tip location. When the axial load exceeds a certain value, which eventually eliminates NSF, NSF need not be taken into account.

## Figures and Tables

**Table 1:** Summary of the elements and nodes in the numerical analyses.

Numerical number	Tip location (*Y*)	Number of mesh		Numerical of nodes	
		Pile	Soil	Pile	Soil
1	1.00*D *	896	8,960	1,189	9,785
2	0.25*D *	928	5,632	1,230	6,373
3	0.00*D *	896	8,960	1,189	9,785
4	−1.00*D *	960	5,440	1,271	6,179

**Table 2:** Constitutive models and material parameters adopted for the numerical analyses.

Solid	Pile	Bottom sand	Clay
Constitutive model	Linear-elastic	Mohr-Coulomb	Cam-clay
*γ*_sat⁡_ (kN/m^3^)	27	19.4	16.3
*ν*	0.18	0.30	0.35
Young's modulus, *E* (kPa)	3 × 10^7^	1.2 × 10^5^	N/A
*M *	N/A	N/A	0.98
*λ*	N/A	N/A	0.14
*κ*	N/A	N/A	0.012
*p*_0_ (kPa)	N/A	N/A	64
*e*_0_	N/A	0.37	1.6
*φ*′	N/A	29.7°	25°
*ψ*	N/A	8.3°	0°
*K*_0_	N/A	0.50	0.58

**Table 3:** Summary of the computed results.

Results	*Y* = 1.00*D*	*Y* = 0.25*D*	*Y* = 0.00*D*	*Y* = −1.00*D*
Maximum dragload (kN)	741 (849*)	907	1571	1390
(Maximum dragload/maximum dragload of pile with *Y* = 1.00*D*) × 100%	100	122	212	188
Neutral plane, *z/L*	0.60 (0.73*)	0.71	0.82	0.75
Maximum soil settlement (mm)	55	75	75	55
Pile capacity (kN)	1080 (1728*)	1625	2822	3955
(Pile capacity/pile capacity of pile with *Y* = 1.00*D*) × 100%	100	150	261	366

*Numbers in brackets denote the measured data.

**Table 4:** Variations of the dragload reduction and base stress increment with the axial load.

Tip location (*Y*)	*P*/*P*_max⁡_																			
	0.25	0.50	0.75	1.00	1.25	1.50	1.75	2.00	2.25	2.50	2.75	3.00	3.25	3.50	3.75	4.00	4.25	4.50	4.75	5.00
*R* (%)																				
*Y* = 1.00	16.2	30.8	45.0	57.1	68.7	79.4	89.2	100	100	100	100	100	100	100	100	100	100	100	100	100
*Y* = 0.25	12.1	23.4	34.2	45.4	56.0	66.7	76.6	85.6	93.7	100	100	100	100	100	100	100	100	100	100	100
*Y* = 0.00	11.7	22.5	31.0	36.9	44.7	52.1	59.3	66.3	73.4	80.2	86.8	92.9	98.9	100	100	100	100	100	100	100
*Y* = −1.00	8.8	16.8	24.2	31.0	37.3	43.2	48.8	54.3	59.9	65.3	70.8	76.0	81.2	86.1	90.7	95.2	99.7	100	100	100
*I* (%)																				
*Y* = 1.00	2.8	5.5	8.0	10.8	12.6	14.9	18.3	21.2	23.9	37.2	56.6	85.5	125	217	310	357	403	449	494	525
*Y* = 0.25	4.5	9.2	14.2	19.4	25.3	31.3	38.4	46.8	56.7	68.1	80.9	95.1	110	127	144	164	187	210	240	272
*Y* = 1.00	10.6	22.5	32.6	40.4	50.3	60.3	70.2	80.3	90.6	100	111	122	134	145	157	170	184	201	221	242
*Y* = 0.25	14.8	28.58	42.08	55.6	69.0	82.6	96.1	110	124	137	150	164	178	190	205	218	233	248	263	279
